# DNA-Binding and Cytotoxicity of Copper(I) Complexes Containing Functionalized Dipyridylphenazine Ligands

**DOI:** 10.3390/pharmaceutics13050764

**Published:** 2021-05-20

**Authors:** Sammar Alsaedi, Bandar A. Babgi, Magda H. Abdellattif, Muhammad N. Arshad, Abdul-Hamid M. Emwas, Mariusz Jaremko, Mark G. Humphrey, Abdullah M. Asiri, Mostafa A. Hussien

**Affiliations:** 1 Department of Chemistry, Faculty of Science, King Abdulaziz University, P.O. Box 80203, Jeddah 21589, Saudi Arabia; salsaedi0029@stu.kau.edu.sa (S.A.); aasiri2@kau.edu.sa (A.M.A.); maabdulaal@kau.edu.sa (M.A.H.); 2 Chemistry Department, Deanship of Scientific Research, College of Sciences, Taif University, Al-Haweiah, P.O. Box 11099, Taif 21944, Saudi Arabia; m.hasan@tu.edu.sa; 3 Center of Excellence for Advanced Materials Research (CEAMR), King Abdulaziz University, P.O. Box 80203, Jeddah 21589, Saudi Arabia; mnarshard@kau.edu.sa; 4 Core Labs, King Abdullah University of Science and Technology (KAUST), Thuwal, 23955-6900, Saudi Arabia; abdelhamid.emwas@kaust.edu.sa; 5 Biological and Environmental Science and Engineering (BESE), King Abdullah University of Science and Technology (KAUST), Thuwal 23955-6900, Saudi Arabia; Mariusz.jaremko@kaust.edu.sa; 6 Research School of Chemistry, Australian National University, Canberra, ACT 2601, Australia; mark.humphrey@anu.edu.au; 7 Department of Chemistry, Faculty of Science, Port Said University, Port Said 42521, Egypt

**Keywords:** Copper(I), dipyridophenazine, DNA-binding, anticancer properties, molecular docking

## Abstract

A set of copper(I) coordination compounds with general formula [CuBr(PPh_3_)(dppz-R)] (dppz-R = dipyrido[3,2-a:2’,3’-c]phenazine (**Cu-1**), 11-nitrodipyrido[3,2-a:2’,3’-c]phenazine (**Cu-2**), 11-cyanodipyrido[3,2-a:2’,3’-c]phenazine (**Cu-3**), dipyrido[3,2-a:2’,3’-c]phenazine-11-phenone (**Cu-4**), 11,12-dimethyldipyrido[3,2-a:2’,3’-c]phenazine (**Cu-5**)) have been prepared and characterized by elemental analysis, ^1^H-NMR and ^31^P-NMR spectroscopies as well as mass spectrometry. The structure of **Cu-1** was confirmed by X-ray crystallography. The effect of incorporating different functional groups on the dppz ligand on the binding into CT-DNA was evaluated by absorption spectroscopy, fluorescence quenching of EtBr-DNA adducts, and viscosity measurements. The functional groups affected the binding modes and hence the strength of binding affinities, as suggested by the changes in the relative viscosity. The differences in the quenching constants (K_sv_) obtained from the fluorescence quenching assay highlight the importance of the functional groups in altering the binding sites on the DNA. The molecular docking data support the DNA-binding studies, with the sites and mode of interactions against B-DNA changing with the different functional groups. Evaluation of the anticancer activities of the five copper compounds against two different cancer cell lines (M-14 and MCF-7) indicated the importance of the functional groups on the dppz ligand on the anticancer activities. Among the five copper complexes, the cyano-containing complex (**Cu-3**) has the best anticancer activities.

## 1. Introduction

**Figure 1:**
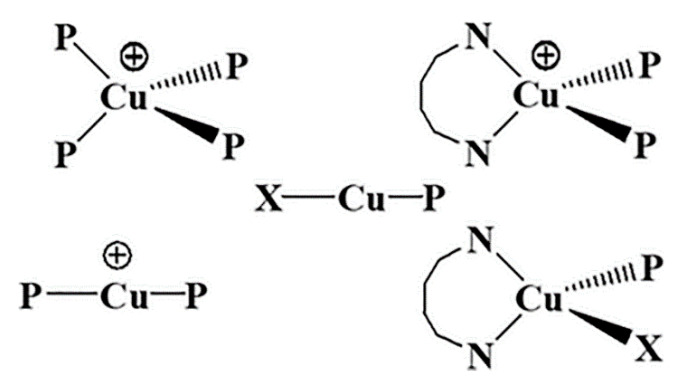
General structures of copper(I) complexes employed in chemotherapy.

The field of metallodrugs has been under scrutiny since the discovery of the anticancer properties of cis-diamminedichloroplatinum(II) (cisplatin). Although cisplatin has been widely applied as a chemotherapeutic agent, it causes severe side effects [1,2,3,4], so there have been intensive efforts to find more effective metal-based anticancer drugs. In the search for new and more effective anticancer drugs, a wide range of transition metal complexes have been synthesized and tested as anticancer agents [5,6]. Copper compounds attracted special interest as possible candidates [7,8,9,10,11] because copper exists in many biological systems and is involved in many biological processes such as energy metabolism and oxygen transportation. In this regard, the interest in copper(I) complexes as potential chemotherapeutic agents have grown enormously in the past three decades (Figure 1) [12]. Recently, a copper(I) phosphine complex, [Cu(PR_3_)_4_][PF_6_] (R = CH_2_OH), has attracted special interest due to its strong anticancer effects. The complex shows particularly strong effects against human colon cancer cells; it is even more efficient than platinum-based drugs and displays minimal effects on non-tumor cells [13,14]. Hydrophilic phosphines such as tris(2-cyanoethyl)phosphine (L1) and bis(2-cyanoethyl)phenylphosphine (L2) were employed in synthesizing the Cu(I) complexes [Cu(L1)_2_]^+^, [Cu(MeCN)(L1)]^+^, [CuX(L1)], and [Cu(L2)_2_]^+^. The new copper(I) complexes were tested for their cytotoxic properties against a suite of human cancer cell lines and were highlighted for their ability to alter the mitochondrial membrane potential and production of reactive oxygen species (ROS) [15]. Recently, complexes were reported with 1,10-phenanthroline (phen) or 2,9-dimethyl-1,10-phenanthroline (dmp) copper(I) iodide complexes and one of three hydrophilic phosphine ligands: P(CH_2_N(CH_2_CH_2_)_2_NCH_3_)_3_, P(CH_2_N(CH_2_CH_2_)_2_O)_3_ and P(CH_2_N(CH_3_)CH_2_CH_2_OH)_3_. The complexes exhibit a strong in vitro antitumor activity against human ovarian carcinoma cell lines (MDAH2774 and SCOV3) with IC_50_ values in the range 2–7 μM [16]. Komarnicka et al. employed a phosphine ligand decorated with a sparfloxacin moiety (an antibiotic agent) in the synthesis of complexes with the general formula [CuXP(NN)]. Evaluation of the in vitro anticancer activity of these complexes against mouse colon carcinoma (CT26) and human lung adenocarcinoma (A549) revealed that the complexes generate reactive oxygen species in the cancer cells, inducing apoptosis and leading to cell death [17]. Despite the negligible cytotoxic effects of varying the diimine ligand in such complexes, diimines have important roles in the complex-DNA interactions. For example, biquinoline-containing complexes are proposed to bind via intercalation, while groove-binding was suggested for the complexes with a 2,9-dimethylphenanthroline ligand [17,18]. Complexes [CuBr(1,10-phenanthroline)(PPh_3_)] and [Cu(1,10-phenanthroline)(P(4-methoxyphenyl)_3_)_2_]^+^ have also been tested against several cell lines and exhibited cytotoxic effects comparable to cisplatin against breast and prostate (MFC-7 and P3) cancer cell lines [19].

**Figure 2:**
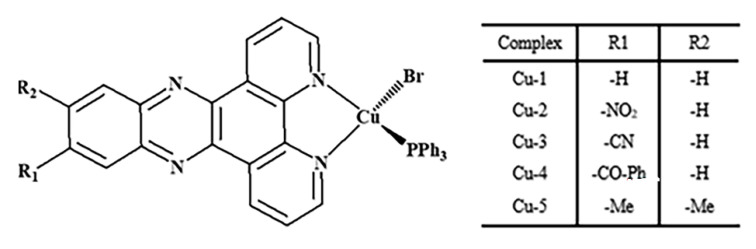
Copper(I) complexes studied in this work.

In recent work, different diimine ligands containing several functional groups were employed in the synthesis of complexes with the general formulation [CuBr(PPh_3_)(diimine)]; optimum cytotoxic effects against prostate (PC-3), leukemia (MOLT-4), and breast (MCF-7) cancer cell lines were observed with ligands containing nitrogen heteroatoms and extended delocalized systems such as dipyrido[3,2-a:2’,3’-c]phenazine (dppz) [20]. Additionally, the DNA-complex binding capability was shown to increase dramatically on employing dppz as a ligand. In this work, we extend our studies to examine the effect of functionalizing the dppz ligands on the antineoplastic and DNA-binding properties of the copper(I) complexes (Figure 2), supporting our experimental observations with molecular docking studies.

## 2. Materials and Methods

### 2.1. Materials

Solvents (HPLC grade) were used without distillation. DNA sodium salt from calf thymus (Type I) was purchased from Sigma-Aldrich (St. Louis, MO, USA). The synthesis of CuBr(PPh_3_)_3_ was achieved as described in the literature, by stirring copper(II) bromide with ca. four molar equivalents of triphenylphosphine in refluxing ethanol under an inert gas atmosphere. The product precipitated after several minutes and was collected by filtration as a white powder [21]. The dipyrido[3,2-a:2’,3’-c]phenazine (dppz) [22], 11-nitro-dipyrido[3,2-a:2’,3’-c]phenazine (dppz-NO_2_) [23], 11-cyano-dipyrido[3,2-a:2’,3’-c]phenazine (dppz-CN) [24], 4,5,9,14-tetraaza-benzo[b]triphenylene-11-yl)-phenyl-methanone (dppz-CO-Ph) [25] and 11,12-dimethyl-dipyrido[3,2-a:2’,3’-c]phenazine (dppz-Me_2_) [26] were prepared by the literature procedure from the reaction of 1,10-phenanthroline-5,6-dione and substituted *o*-phenylenediamine in refluxing ethanol. The synthesis of CuBr(PPh_3_)(dppz) (**Cu-1**) was synthesized by stirring equimolar amounts of CuBr(PPh_3_)_3_ and dppz in dichloromethane as described in the literature [19].

### 2.2. Methods and Instrumentation

Infrared spectra were collected for powder samples of the complexes, using a Bruker Alpha FT-IR; bands are reported in cm^−1^. ^1^H NMR (600 MHz) and ^31^P NMR (242 MHz) spectra were collected by a Bruker Avance 600 MHz spectrometer furnished with a BBO probe (BrukerBioSpin, Rheinstetten, Germany). The spectra were collected using parameters described in the literature [27]. High-resolution electrospray ionization (HR ESI) mass spectra were collected by an Agilent Q-TOF 6520 (Shelton, CT, USA) instrument using acetonitrile solutions of the compounds; all mass spectrometric data are listed as *m*/*z*. Electronic spectra were obtained by a Genesys-10s UV-VIS spectrophotometer (Thermo Fischer Scientific, Waltham, MA, USA), using 1 cm path-length quartz cells; the absorption maxima are reported in the form of wavelength in nanometer.

### 2.3. Synthesis and Characterizations

#### General Procedure for the Synthesis of Copper(I) Complexes

CuBr(PPh_3_)_3_ and equimolar amount of the dppz ligand were mixed under nitrogen in a suitable amount of dichloromethane. The colorless solution became orange in a few minutes and was stirred for 2 h. The resulted solution was then reduced in volume and 40 mL petroleum spirit (40–60 °C) was added, causing to the precipitation of the product. The product was gathered by filtration, rinsed with diethylether, and dried.

CuBr(PPh_3_)(dppz-NO_2_) (**Cu-2**). Dppz-NO_2_ (0.070 g, 0.214 mmol) and CuBr(PPh_3_)_3_ (0.198 g, 0.214 mmol) reacted to afford **Cu-2** as an orange powder (0.116 g, 73%). IR (solid, cm^−1^): 500 ν(Cu-N), 522 ν(P-Cu), 1522 ν(aromatic C=C), 1578 ν(aromatic C=N). ^1^H NMR (CDCl_3_, 600 MHz): δ 9.63 (br s, 1H, dppz), 9.26 (br s, 2H, dppz), 9.08 (br s, 1H, dppz), 8.70 (d, J_HH_ = 6 Hz, 2H, dppz), 8.51 (d, J_HH_ = 6 Hz, 2H, dppz), 7.87 (br s, 1H, dppz), 7.52 (br s, 6H, PPh_3_), 7.32 (m, 3H, PPh_3_), 7.27 (m, 6H, PPh_3_). ^31^P {^1^H} NMR (CDCl_3_, 600 MHz): −1.56 (s, PPh_3_). HR ESI MS: calcd for [C_36_H_23_^79^Br^63^CuN_5_O_2_P]^+^ [M-H]^+^ 730.00633, found 730.00589; calcd for [C_36_H_24_^63^CuN_5_O_2_P]^+^ [M-Br]^+^ 652.09581, found 652.09518. ESI MS: 652.09518 [Cu(PPh_3_)(dppz-NO_2_)]^+^, 587.11074 [Cu(PPh_3_)_2_]^+^, 431.03081 [Cu(dppz-NO_2_)(MeCN)]^+^, 366.04648 [Cu(PPh_3_)(MeCN)]^+^. Anal. Calcd for C_36_H_24_BrCuN_5_O_2_P: C, 58.99; H, 3.30; N, 9.55%; found: C, 59.43; H, 3.67; N, 9.26%.

CuBr(PPh_3_)(dppz-CN) (**Cu-3**). Dppz-CN (0.100 g, 0.326 mmol) and CuBr(PPh_3_)_3_ (0.303 g, 0.326 mmol reacted to yield **Cu-3** as an orange powder (0.117 g, 50%). IR (solid, cm^−1^): 500 ν(Cu-N), 522 ν(P-Cu), 2228 ν(CN), 1576 ν(aromatic C=C), 1655 ν(aromatic C=N), 2228 ν(CN).^1^H NMR (CDCl_3_, 600 MHz): δ 9.55 (br s, 2H, dppz), 9.08 (br s, 2H, dppz), 8.72 (s, 1H, dppz), 8.43 (d, J_HH_ = 6 Hz, 1H, dppz), 8.06 (d, J_HH_ = 12 Hz, 1H, dppz), 7.85 (br s, 2H, dppz), 7.50 (br s, 6H, PPh_3_), 7.32 (m, 3H, PPh_3_), 7.27 (m, 6H, PPh_3_). ^31^P {^1^H} NMR (CDCl_3_, 600 MHz): −1.68 (s, PPh_3_). HR ESI MS: calcd for [C_37_H_23_^79^Br^63^CuN_5_P]^+^ [M-H]^+^ 710.01650, found 710.01545; calcd for [C_37_H_24_^63^CuN_5_P]^+^ [M-Br]^+^ 632.10598, found 632.10480. ESI MS: 632.10480 [Cu(PPh_3_)(dppz-CN)]^+^, 587.11034 [Cu(PPh_3_)_2_]^+^, 411.04070 [Cu(dppz-CN)(MeCN)]^+^, 366.04620 [Cu(PPh_3_)(MeCN)]^+^. Anal. Calcd for C_37_H_24_BrCuN_5_P: C, 62.32; H, 3.39; N, 9.82%; found: C, 61.96; H, 3.52; N, 9.47%.

CuBr(PPh_3_)(dppz-CO-Ph) (**Cu-4**). Dppz-CO-Ph (0.030 g, 0.079 mmol) and CuBr(PPh_3_)_3_ (0.074 g, 0.079 mmol) reacted to yield **Cu-4** as an orange powder (0.045 g, 72%). IR (solid, cm^−1^): 500 ν(Cu-N), 522 ν(P-Cu), 1573 ν(aromatic C=C), 1594 ν(aromatic C=N), 1655 ν(aromatic C=O). ^1^H NMR (CDCl_3_, 600 MHz): δ 9.64 (br s, 1H, dppz), 9.57 (br s, 1H, dppz), 9.06 (br s, 2H), 8.70 (s, 1H, dppz), 8.48 (d, J_HH_ = 12 Hz, 1H, dppz), 8.41 (d, J_HH_ = 6 Hz, 1H, dppz), 7.96 (d, J_HH_ = 6 Hz, 2H, dppz), 7.85 (d, J_HH_ = 7 Hz, 2H, COPh), 7.69 (t, J_HH_ = 7 Hz, 1H, COPh), 7.58 (t, J_HH_ = 7 Hz, 2H, COPh), 7.49 (br s, 6H, PPh_3_), 7.32 (m, 3H, PPh_3_), 7.27 (m, 6H, PPh_3_). ^31^P {^1^H} NMR (CDCl_3_, 600 MHz): −1.92 (s, PPh_3_). HR ESI MS: calcd for [C_43_H_29_^63^CuN_4_OP]^+^ [M-Br]^+^ 711.13750, found 711.13554. ESI MS: 711.13554 [Cu(PPh_3_)(dppz-CO-Ph)]^+^, 587.11007 [Cu(PPh_3_)_2_]^+^, 490.07134 [Cu(dppz-CO-Ph)(MeCN)]^+^, 366.04605 [Cu(PPh_3_)(MeCN)]^+^. Anal. Calcd for C_43_H_29_BrCuN_4_OP: C, 65.20; H, 3.69; N, 7.07%; found: C, 64.63; H, 3.22; N, 6.68%.

CuBr(PPh_3_)(dppz-Me_2_) (**Cu-5**). DPPZ-Me_2_ (0.070 g, 0.225 mmol) and CuBr(PPh_3_)_3_ (0.210 g, 0.225 mmol) reacted to yield **Cu-5** as an orange powder (0.102 g, 64%). IR (solid, cm^−1^): 500 ν(Cu-N), 522 ν(P-Cu), 1477 ν(aromatic C=C), 1573 ν(aromatic C=N).^1^H NMR (CDCl_3_, 600 MHz): δ 9.59 (br s, 2H, dppz), 9.02 (br s, 2H, dppz), 8.09 (br s, 2H, dppz), 7.79 (br s, 2H, dppz), 7.49 (br s, 6H, PPh_3_), 7.25 (br s, 9H, PPh_3_), 2.61 (s, 6H, CH_3_). ^31^P {^1^H} NMR (CDCl_3_, 600 MHz): −2.39 (s, PPh_3_). HR ESI MS: calcd for [C_38_H_29_N_4_CuP]^+^ [M-Br]^+^ 635.14258, found 635.14121. ESI MS: 635.14121 [Cu(PPh_3_)(dppz- Me_2_)]^+^, 587.11007 [Cu(PPh_3_)_2_]^+^, 366.04605 [Cu(PPh_3_)(MeCN)]^+^. Anal. Calcd for C_38_H_29_BrCuN_4_P: C, 63.74; H, 4.08; N, 7.82%; found: C, 63.61; H, 4.97; N, 7.64%.

### 2.4. Crystal Structure Determination

Crystals of **Cu-1** suitable for structural studies were achieved by vapor diffusion of hexane into a solution of the compound in dichloromethane at 5 °C. Crystals were detected under a microscope and an appropriate crystal was selected and mounted on an Agilent SuperNova (dual source) Agilent Technologies Diffractometer (Agilent technologies, Santa Clara, CA, USA), supplied with microfocused Cu/Mo Kα radiation for data collection. The data collection was undertaken using CrysAlisPro software [28] at 296 K with Mo Kα radiation. The structure solution was completed using SHELXS–97 [29] and refined by full–matrix least-squares methods on F2 using SHELXL–97 [29], interfaced with WinGX [30]. All non–hydrogen atoms were processed anisotropically by full–matrix least-squares methods [29]. The figures were created through PLATON [31] and ORTEP [32] interfaced with WinGX. All hydrogen atoms were placed geometrically and treated as riding atoms with C–H = 0.93 Å and Uiso(H) = 1.2Ueq(C) for all carbon atoms. The deposition number 205847 at the Cambridge Crystallographic Data Center contains the full crystal data of **Cu-1**. Crystal data can be obtained free of charge from CCDC, 12 Union Road, Cambridge CB21 EZ, UK (Fax: (+44) 1223 336-033; e-mail: data_request@ccdc.cam.ac.uk).

### 2.5. DNA-Binding Studies

#### 2.5.1. Spectroscopic Determination of Binding Affinities

Absorption spectra of the complexes in the presence of calf thymus DNA were recorded, subtracting the DNA absorption. Six solutions in 5 mM Tris-HCl (pH = 7.4) and 50 mM NaCl were prepared by maintaining the concentration of copper complexes at 20 µM (5% *v*/*v* DMSO was used to enhance the solubility of the complexes as they are not soluble in aqueous mediums) and varying the ratio of [DNA]/[Cu complex] (from 0 to 8.0). To compare the quantitative affinity of the complexes bound to DNA, the intrinsic-binding constant K_b_ can be calculated from the following Equation (1) based on the titration process:

([DNA])/((εA − εF)) = ([DNA])/((εB − εF)) + 1/K_b_ (εB − εF) (1)

where εA, εB and εF correspond to the extinction coefficient for the copper complex at the specific addition of DNA, before the addition of DNA, and at the fully bound mode, respectively. K_b_ is obtainable from plotting [DNA]/(εA − εF) versus [DNA] [33].

#### 2.5.2. Ethidium Bromide Fluorescence Quenching

A solution containing 100 µM ct-DNA and 10 µM ethidium bromide (EtBr) was prepared in aqueous Tris-HCl/NaCl buffer system (pH = 7.2). The ct-DNA-EtBr solutions were incubated for at least 24 h. The solution was treated with different concentrations of each one of the copper complexes in DMSO (the amount of DMSO was ca. 5% *v*/*v*), maintaining the ct-DNA and EtBr concentrations. The changes in the emission spectra of the solutions were followed between 500 nm and 750 nm upon excitation at 390 nm after incubating the complexes for four min. The Stern-Volmer quenching constants (K_SV_) were calculated using the Equation (2) below:

F_o_/F = 1 + K_sv_ [Cu complex] (2)

where F_o_ and F are the emission intensities in the absence and the presence of the samples, respectively. The [Cu complex] was plotted against [F_o_/F]; the K_SV_ value was equal to the slope [33,34].

#### 2.5.3. Determination of Binding Mode by Viscometry

The viscosity measurements were conducting using an Ostwald viscometer. Micro volumes (10 µL) of copper complexes were added to a solution of ct-DNA in buffer where the [Cu complex]/[DNA] ratio was maintained in the range 0.02 to 0.2. The solutions were allowed to incubate for four min at 25 °C in a water bath before measurements. The flow times for the solutions were recorded and replicated at least four times. The relative viscosity (η/η_o_)^1/3^ were plotted against [Cu complex]/[DNA], where η_o_ and η represent the specific viscosity of the ct-DNA alone and the ct-DNA-Cu, respectively. The specific viscosity η and η_o_ were calculated using the formulation [(t − t_b_)/t_b_] where *t* is the observed flow time and *t_b_* is the buffer flow time [33,34].

### 2.6. Molecular Docking Studies

Molecular Operating Environment (MOE) 2008.10 software was employed to conduct molecular docking studies. The docking scores were first achieved, operating the London dG function in the MOE; scores optimizations were proceeded by another two unrelated refinement methods. Grid-Min pose and Force-field were employed to confirm that the refined poses of the complexes were geometrically correct. Geometries around the copper atoms and the aromatic systems planarity were constrained while allowing bond rotations for other groups; the best five binding poses were then examined. To assess the binding free energy of the complexes toward DNA, the docking poses of the complexes and the co-crystallized structure of the B-DNA were docked (RSCP PDB code: 1BNA). Assessment of the best binding pose was achieved by selecting poses that involve interactions with nucleobases and have the lowest RMSD value.

### 2.7. Anticancer Studies

The cells were supplied by the Egyptian Holding Company for Biological Products and Vaccines (VACSERA) and then kept in the tissue culture unit. The growth of the cells was proceeded in RPMI-1640 medium, supplemented with FBS (10% heat inactivated), penicillin (50 units/mL), and streptomycin (50 mg/mL), and kept in a humidified atmosphere with 5% carbon dioxide [35,36]. The cells were kept as monolayer cultures by serial sub-culturing, obtaining cell culture reagents from Lonza (Basel, Switzerland). The antitumor activities of the complexes were assessed against MCF-7 (breast) and M-14 (Melanoma) cell lines.

The sulforhodamine B (SRB) assay method was applied to determine the cytotoxicity, as described in the literature [37]. Exponentially growing cells were gathered using 0.25% Trypsin-EDTA and seeded in 96-well plates at 1000–2000 cells/well in RBMI-1640 supplemented medium. The cells were maintained in the medium for 1 day and then they were incubated for 3 days with various concentrations of the tested complexes. Following 3 days treatments, the cells were fixed with 10% trichloroethanoic acid for 1 h at 4 °C. Wells were stained for 10 min at room temperature with 0.4% SRBC dissolved in 1% acetic acid. The plates were air dried for 24 h and the dye was dissolved in Tris-HCl for 5 min with shaking at 1600 rpm. The optical density (OD) of each well was assessed spectrophotometrically at 564 nm with an ELISA microplate reader (ChroMate-4300, FL, USA). The IC_50_ values were calculated from a Boltzmann sigmoidal concentration response curve using the nonlinear regression fitting models (Graph Pad, Prism Version 9).

## 3. Results and Discussion

### 3.1. Synthesis and Characterization

**Scheme 1:**
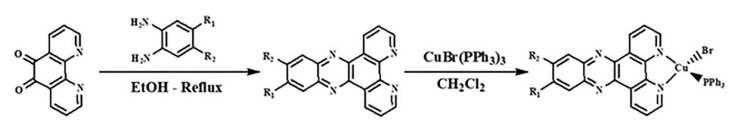
Synthetic route to the copper(I) complexes.

The functionalized dppz ligands were synthesized in good yields by refluxing 1,10-phenanthroline-5,6-dione and the corresponding 1,2-diaminobenzene in ethanol solutions. Copper(I) complexes were synthesized as depicted in Scheme 1, by reacting stoichiometric amounts of the dppz derivatives and CuBr(PPh_3_)_3_ under inert conditions; the complexes were obtained in moderate to good yields. All synthesized complexes in this work are air and moisture stable in the solid state, and there is no sign of degradation while storing the complexes at room temperature for several months. The identities of the complexes were confirmed by ^1^H and ^31^P NMR spectroscopies as well as elemental analysis and mass spectrometry (Figures S1–S12). In the ^31^P NMR spectra, the compounds show an intense singlet peaks around −2 ppm. The mass spectra afford molecular ions in the case of **Cu-1**, **Cu-2** and **Cu-3** while the ion [M-Br]^+^ was detected for all complexes. Elemental analysis provided data that are an agreement with the proposed formula. **Cu-1** was subjected to a single-crystal X-ray diffraction study. The five copper complexes have shown stability at ambient conditions as solids, DMSO solutions and acetonitrile solutions.

### 3.2. Crystallographic Studies

**Figure 3:**
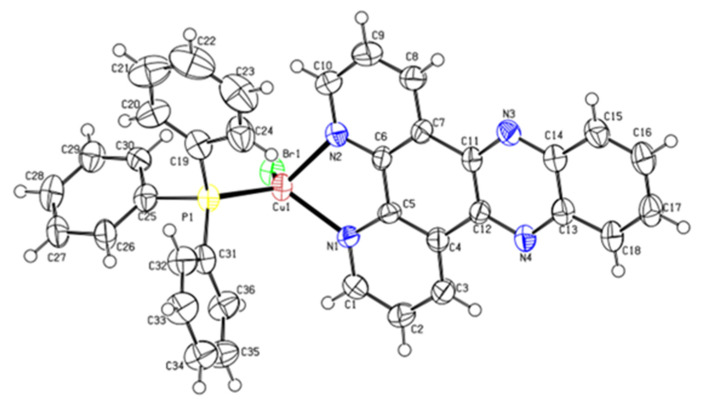
ORTEP diagram of **Cu-1** with thermal ellipsoids drawn at the 50% probability level.

**Figure 4:**
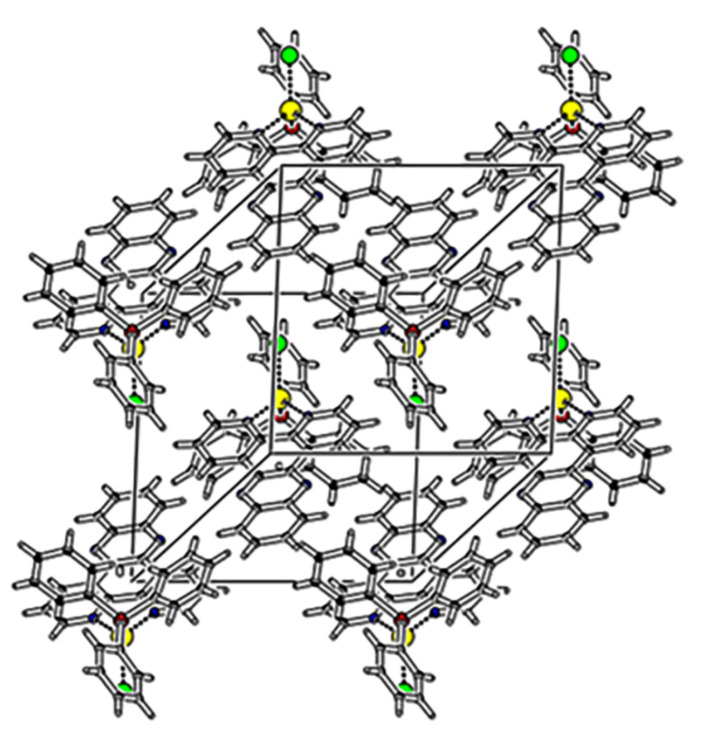
Unit cell packing diagram for **Cu-1**.

The structure of **Cu-1** is illustrated in Figure 3 and a unit cell packing view is shown in Figure 4. Very thin and small needles of **Cu-1** were obtained, resulting in a structure solution with considerable disorder in the solvent molecules within the crystal. The Cu atom is coordinated by four atoms (P1, Br1, N1 and N2) and adopts a pseudotetrahedral geometry. The geometry is more precisely trigonal pyramidal, considering that the geometry index (τ_4_) is 0.847 [19,20]; the geometry index is obtained from the formula τ_4_ = [360 − (α + β)]/141, where α and β are the largest angles around the Cu-metal atom. The value of τ_4_ should be one for tetrahedral and zero for square planar [38]. The angles around the Cu atoms are 122.77(18)°, 117.72(6)°, 113.12(18)°, 112.12(17)°, 105.60(17)° and 78.92(0.23)°. The plane produced from the fitted atoms of the dppz ligand is not perfectly planar, as the observed root mean square (r.m.s.) deviation for this plane is 0.0506(7)Å, where the major deviations (0.0683(8) Å and −0.1023(7) Å) are observed with the C15 and C10 atoms, respectively. A five-membered ring motif (Cu1/N1/C5/C6/N2) produced through the coordination via the nitrogen atoms of the dppz ligand with the central Cu atom has an r.m.s. deviation of 0.0370 Å. The plane produced from the atoms of the dppz ligand is twisted by 2.915(3)° with respect to the five-membered ring motif (Cu1/N1/C5/C6/N2). The observed puckering parameters for this ring motif are Q2 = 0.086(6)Å and φ = 161(5)°. No hydrogen bonding is seen among the molecules in the unit cell, but there are π-π interactions among the planes of molecules (Figure 4). The plane produced from the atoms (C4/C5/C6/C7/C11/C12) interacts with the plane of (C13/C14/C15/C16/C17/C18) at an angle of 2.252°, with a centroid-centroid distance of 3.493 Å, while the centroid distance between the planes (C13/C14/C15/C16/C17/C18) and (N1/C1/C2/C3/C4/C5) is 3.860 Å with an interplanar angle of 1.224°.

### 3.3. Absorption and Emission Spectra

**Figure 5:**
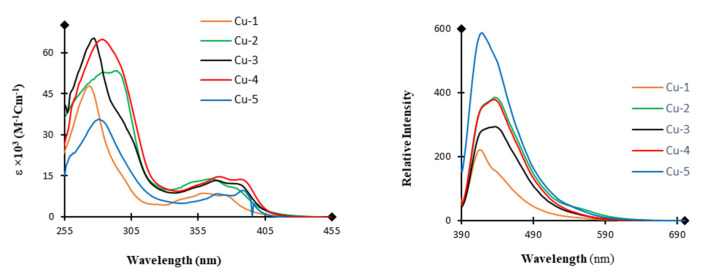
Overlaid absorption (left) and emission (right) spectra of complexes **Cu-1**–**Cu-5**.

The electronic absorption spectra of the five complexes were recorded in DMSO solutions and are shown in Figure 5 (left). The spectra of complexes exhibit two major bands: intense bands in the range 255 to 315 nm assigned as LC (π–π*) and other broad bands in the range 330 to 415 nm attributed to ^1^MLCT (d- π*) with a contribution from intra-ligand transitions in the diimine ligands [39,40]. The molar absorptivity of λ_max_ for the LC transitions are in the range (35.82–65.36) × 10^3^ M^−1^ cm^−1^, while for the lower energy MLCT bands, extinction coefficients are in the range (8.54–13.85) × 10^3^ M^−1^ cm^−1^ (Table 1). The emission spectra of the complexes (Figure 5 (right)) were measured in deoxygenated DMSO solutions with 5 × 10^−5^ M concentrations, and all samples being excited at 370 nm; the excitation wavelength was selected experimentally by scanning in the proximity of the bands in the absorption spectra. The emission wavelengths (λ_em_) observed (416, 418, 436, 437, and 437 nm for **Cu-1**, **Cu-5**, **Cu-2**, **Cu-3**, and **Cu-4**, respectively); red shift of ca. 19 nm was noted upon introduction of withdrawing groups as in proceeding from **Cu-1** to **Cu-2**, **Cu-3** or **Cu-4** (Table 2). The trend in increasing relative intensity (**Cu-1** < **Cu-3** < **Cu-2** ≅ **Cu-4** < **Cu-5**) corresponds with increasing bulkiness; the two methyl groups reduce the quenching by π-π interactions.

### 3.4. DNA-Binding Studies

**Figure 6:**
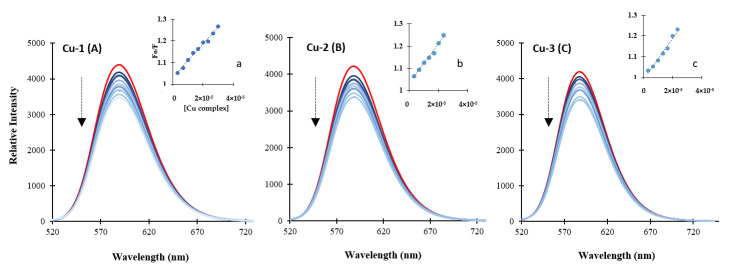
Quenching of EtBr-DNA adduct by **Cu-1** (**A**), **Cu-2** (**B**), and **Cu-3** (**C**) (red solid lines are emission spectra of the EtBr-DNA adduct, while the blue lines are the emission spectra with increasing concentrations of the complexes. (a,b,c) Stern-Volmer plots of the quenching process of EtBr-DNA by **Cu-1**, **Cu-2**, and **Cu-3**.

Due to the insolubility of our complexes in aqueous medium, minimum amount of DMSO was used to prepare solutions for biological studies. The stabilities of complexes were evaluated in DMSO and 20% DMSO in buffer and the complexes exhibited stability as indicated by UV-vis absorption over days without any sign of degradation. Copper(I)-phosphine complexes show two absorption bands in the UV/Vis spectral range, both possessing charge-transfer character and both involving the π-orbitals of the dppz ligands. The interaction of the aromatic system of the ligand with the nucleobase pairs may lead to a decrease in the HOMO-LUMO energy gap (a bathochromic shift) with a decrease in the band intensities (a hypochromic shift). The intrinsic-binding constants (K_b_) obtained above deliver information on the strength of the binding with the DNA which results from the different interaction modes (Table 2). **Cu-2** and **Cu-1** have similar strengths in their binding toward ct-DNA while **Cu-3** and **Cu-4** exhibit only 20% of the binding strength observed in **Cu-1** and **Cu-2**. **Cu-5** exhibits the weakest binding (5% of that of **Cu-1**). Structure-binding correlations for the complexes have also been explored by a fluorescence quenching assay (Table 2). Molecules that normally have a higher ability to intercalate to DNA would eject ethidium bromide from the DNA. Our Cu(I) complexes are equipped with the strongly intercalating ligand dppz and its derivatives. The emissive EtBr-DNA adduct was treated with varying concentrations of the complexes which compete with the ethidium bromide for binding to DNA and thereby act as emission-quenchers (Figure 6). All five complexes partially quench the EtBr-DNA system but to varying extents (Table 2). **Cu-3** shows the largest quenching value (K_SV_), 1.00 × 10^4^ M^−1^. Although EtBr is a well-established intercalative binder, the displacing molecule is not necessarily an intercalator itself, since the only requirement is that it binds to DNA more firmly than EtBr in the same site or around it (between two A-T pairs). Thus, this assay does not provide conclusive information on the binding mode, but it does confirm the strength of the interaction.

**Figure 7:**
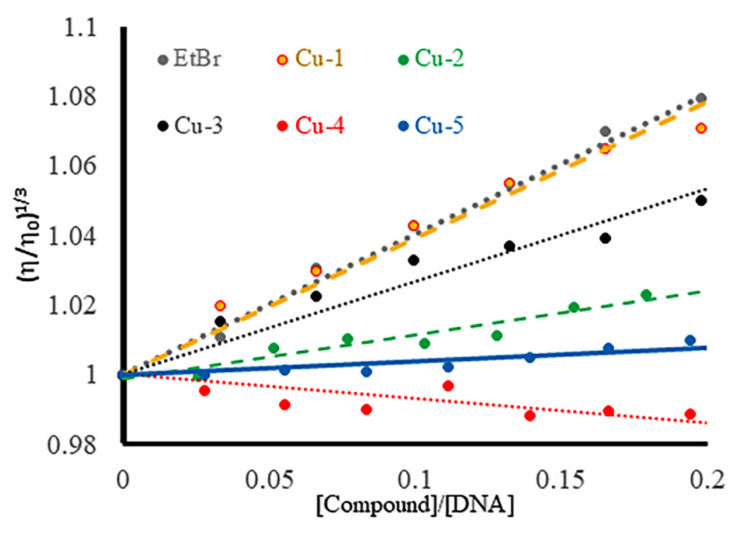
Determination of the binding modes of the compounds to DNA by viscosity assay.

The trend in the changes of the viscosity of DNA can suggest the types of interaction with DNA that the complexes undergo. For instance, if no effect on the relative viscosity of the DNA solution is observed, the compound interacts electrostatically [41]. Similarly, groove-binders do not change the viscosity of the DNA. On the other hand, intercalative binding causes an increase in viscosity due to the elongation of the DNA polymer [42]. The partial intercalation of a molecule leads to bending (kinking) of the DNA chain, decreasing its viscosity [43]. Covalently bound molecules cause unwinding of the DNA double helix, decreasing the relative viscosity of the DNA solution [44]. We undertook viscosity measurements of our complexes and the typical intercalator ethidium bromide (Figure 7). Our systems have at least one more binding mode added to the typical intercalation, originating from the substituent on the dppz ligand. Ethidium bromide is a well-known DNA binder exclusively through intercalation. Hence, we used the viscosity change of ethidium bromide as a benchmark for intercalation. From the curve of the change of the relative viscosity, we can assume **Cu-1** has at least a similar intercalating ability. Based on that assumption, and considering the intrinsic-binding constant, **Cu-2** has similar binding results that accumulate from different binding modes. The change in relative viscosity of the DNA upon additions of **Cu-2** suggests intercalation (increases the viscosity) and partial intercalation (decreases the viscosity) modes of binding. Other modes of binding such as electrostatic interactions are possible as well. **Cu-3** has better intercalation ability than that exhibited by **Cu-2** and/or weaker partial intercalation, which leads to a smaller intrinsic-binding constant. **Cu-4** is partially intercalative, as is evident from the decrease in relative viscosity; the large benzoyl substituent prevents the extended planar ligand system from intercalating inside the minor groove and interacting with the nucleobase pairs. **Cu-5** does not cause any pronounced change in the viscosity, suggesting no intercalation interactions.

### 3.5. Molecular Docking

**Figure 8:**
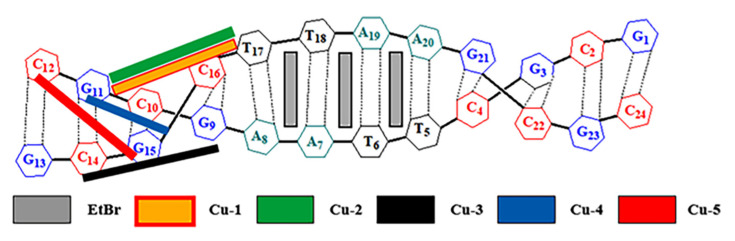
Cartoon illustration of the possible binding sites of the Cu-compounds as estimated by molecular docking modeling.

Docking studies were conducted to highlight the possible sites of interactions and the different modes of interactions, to help rationalize the binding information obtained by the fluorescence quenching and viscosity measurements. The sequence of the docked B-DNA is shown in Figure 7, with a representative illustration of binding sites of EtBr, **Cu-1**, **Cu-2**, **Cu-3**, **Cu-4**, and **Cu-5** as obtained by the docking study. The binding sites of **Cu-1**, **Cu-2**, and **Cu-3** approach the potential binding sites of ethidium bromide, which rationalizes their greater displacements in the fluorescence quenching assay (Figure 8).

**Figure 9:**
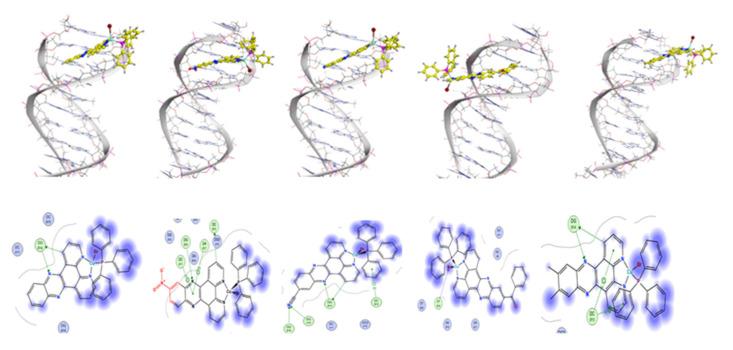
Illustration of the non-covalent interactions (2D and 3D) of Cu-compounds with B-DNA as estimated by MOE.

All the complexes can establish different interactions. However, complexes **Cu-1**, **Cu-2**, and **Cu-3** favor side intercalative interactions between the G-C nucleobase pairs (Figure 8). In Figure 9, the 2-D illustrations show the areas that are exposed (outside the DNA) colored in blue while the uncolored areas indicate the regions sandwiched between the DNA strands. **Cu-4** and **Cu-5** stack in the major grooves on one of the DNA strands, which can be explained by the steric bulkiness of their substituents (Figure 9).

### 3.6. Anticancer Studies

The five copper complexes and cisplatin (as a positive control) were examined against two cancer cell lines: melanoma skin cell line (M-14) and the hormone-dependent breast cancer cell line (MCF-7). In our previous work, we concluded that the presence of highly π-delocalized planar diimine ligands such as dppz in CuBr(PPh_3_)(diimine) helps to improve the anticancer activities. In this manuscript, the effect of functionalizing such ligands has been examined. Adding functional groups such as nitro, cyano, benzoyl, and methyl enhances the cytotoxicity to different extents (Table 3). The cyano group seems to be the most influential, improving the IC_50_ by 40% (against MCF-7) and 60% (against M-14). The benzoyl and nitro groups cause similar cytotoxic effects while the methyl groups cause less pronounced cytotoxic effects. Moreover, two of the ligands (dppz-CN and dppz-CO-Ph) were examined against M-14 cell line to validate the role of the ligand in the anticancer properties of the copper(I) complexes. The ligands show low cytotoxicity when compared to their copper complexes, confirming the role of the metal center in the anticancer properties. Extracting simple structure-cytotoxic correlations is not straightforward and requires a good understanding of the activation mechanism, and small structural modifications could change the mechanism of action of the complexes, as seen previously with other copper complexes [45]. Further studies are needed to highlight the proper role of the cyano group in improving anticancer activities.

## 4. Conclusions

In the current work, four new copper complexes containing different functionalized dppz ligands were synthesized and characterized by spectroscopic techniques. CuBr(PPh_3_)(dppz) (**Cu-1**) was further characterized by X-ray diffraction techniques. The effects of incorporating different functional groups (nitro in **Cu-2**, cyano in **Cu-3**, benzoyl in **Cu-4**, and dimethyl in **Cu-5**) on the binding into CT-DNA were evaluated by absorption spectroscopy, fluorescence quenching of the EtBr-DNA adduct, and viscosity measurements. The changes in the relative viscosity suggest that the functional groups affect the binding modes and hence the strength of binding affinities. Moreover, the fluorescence quenching assay indicates that the functional groups alter the site of binding. The molecular docking studies support the DNA-binding studies, with the sites and modes of interactions against B-DNA changing with the different functional groups. Anticancer activities of the five copper compounds were evaluated against two different cancer cell lines (M-14 and MCF-7), benchmarking their performance against the well-known chemotherapeutic drug cisplatin. Dramatic changes were observed, highlighting the important rule of the functional groups on the dppz ligand. Among the five copper complexes, **Cu-3** has the optimum anticancer activities, attributed to the presence of the cyano group. The trend among the complexes in anticancer properties is following the same trend in the increase in quenching constant of EtBr-DNA adduct. Further studies are needed to evaluate the actual mechanism of action in **Cu-3** to understand the contributing factors in the cyano group to the anticancer activities.

## Figures and Tables

**Table 1:** Spectroscopic Data of Complexes **Cu-1**–**Cu-5**.

Complex	Absorbance		Emission	
	λ_max_ (nm)	ϵ (10^3^ M^−1^ cm^−1^)	λ_em_, (nm)	Relative Intensity
**Cu-1**	273 359	47.69 8.61	416	0.38
**Cu-2**	289 364	52.67 13.89	436	0.66
**Cu-3**	277 368	65.36 13.39	437	0.50
**Cu-4**	283 370 387	64.98 14.86 13.85	437	0.64
**Cu-5**	281 369 388	35.82 8.55 9.68	418	1.00

**Table 2:** Intrinsic-Binding Constants and Competitive Quenching Constants for **Cu-1**–**Cu-5**.

Complex	Substituent	UV-vis Spectroscopic Titration		Fluorescence Quenching Assay	
		K_b_	Scaled K_b_	K_sv_	Scaled K_sv_
**Cu-1**	H	4.00 × 10^6^	20	7.73 × 10^3^	1.00
**Cu-2**	NO_2_	4.44 × 10^6^	22	8.93 × 10^3^	1.16
**Cu-3**	CN	8.00 × 10^5^	4	1.00 × 10^4^	1.29
**Cu-4**	Benzoyl	8.00 × 10^5^	4	5.53 × 10^3^	0.72
**Cu-5**	Dimethyl	2.00 × 10^5^	1	6.36 × 10^3^	0.82

K_sv_ = quenching constant, K_b_ = intrinsic-binding constant.

**Table 3:** Anticancer Activities of the Copper Complexes and Cisplatin Against M-14 and MCF-7 Cell Lines.

Compounds	IC_5__0_ (µM)	
	M-14	MCF-7
**cisplatin**	6.29 ± 0.05	8.69 ± 0.43
**Dppz-NO_2_**	57.64 ± 0.05	-
**Dppz-CN**	45.20 ± 0.05	-
**Cu-1**	22.82 ± 1.04	11.93 ± 0.54
**Cu-2**	13.83 ± 0.46	9.85 ± 0.10
**Cu-3**	9.14 ± 0.05	7.33 ± 0.38
**Cu-4**	12.88 ± 0.09	8.43 ± 0.62
**Cu-5**	14.76 ± 0.31	11.52 ± 0.52

## Data Availability

The data presented in this study are available on request from the authors.

## Supplementary Materials

The following are available online at https://www.mdpi.com/article/10.3390/pharmaceutics13050764/s1, Figures S1–S4: ^1^H NMR data; Figures S5–S8: ^31^P NMR data; Figures S9–S12: Mass spectra data.

## Abbreviations

Dppz	dipyrido[3,2-a:2’,3’-c]phenazine.
EtBr	Ethidium bromide.
M14	melanoma skin cancer cell line.
MCF-7	breast cancer cell line.
