# Supplementary material for: DNA-Binding and Cytotoxicity of Copper(I) Complexes Containing Functionalized Dipyridylphenazine Ligands
Source: Pharmaceutics. 2021 May 20;13(5):764. doi: 10.3390/pharmaceutics13050764 (PMC8161420; doi:10.3390/pharmaceutics13050764)
Supplement: Supplementary file 1 [file pharmaceutics-13-00764-s001.zip › pharmaceutics-1195206 sup for xml.pdf]

pharmaceutics

MDPI

Supplementary Materials: DNA-Binding and Cytotoxicity of Copper(I) Complexes Containing Functionalized Dipyridylphenazine Ligands

Sammar Alsaedi, Bandar A. Babgi, Magda H. Abdellattif, Muhammed N. Arshad, Abdul-Hamid M. Emwas, Mariusz Jaremko, Mark G. Humphrey, Abdullah M. Asiri and Mostafa A. Hussien

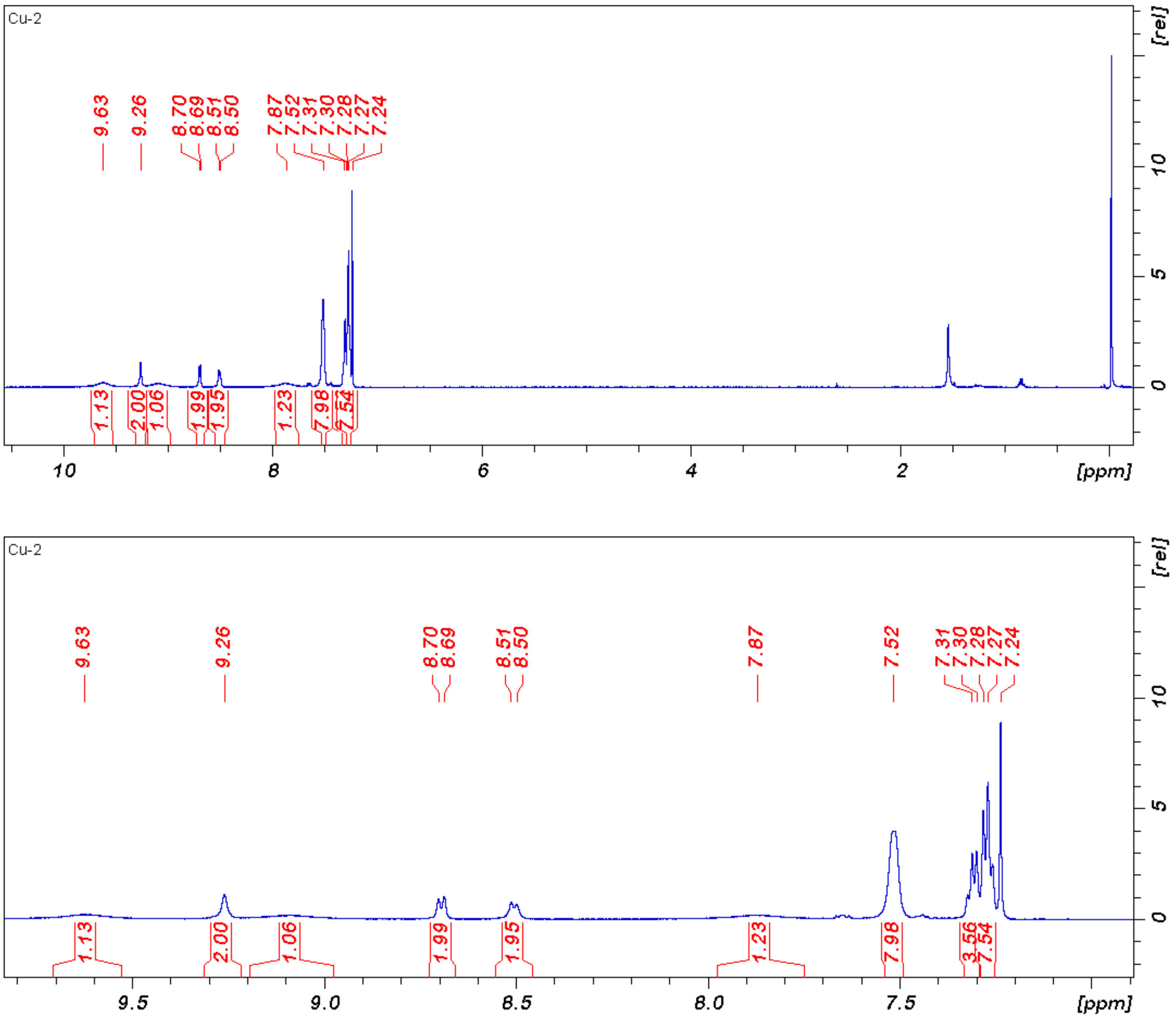

Figure S1. ^{1}H NMR of complex Cu-2.

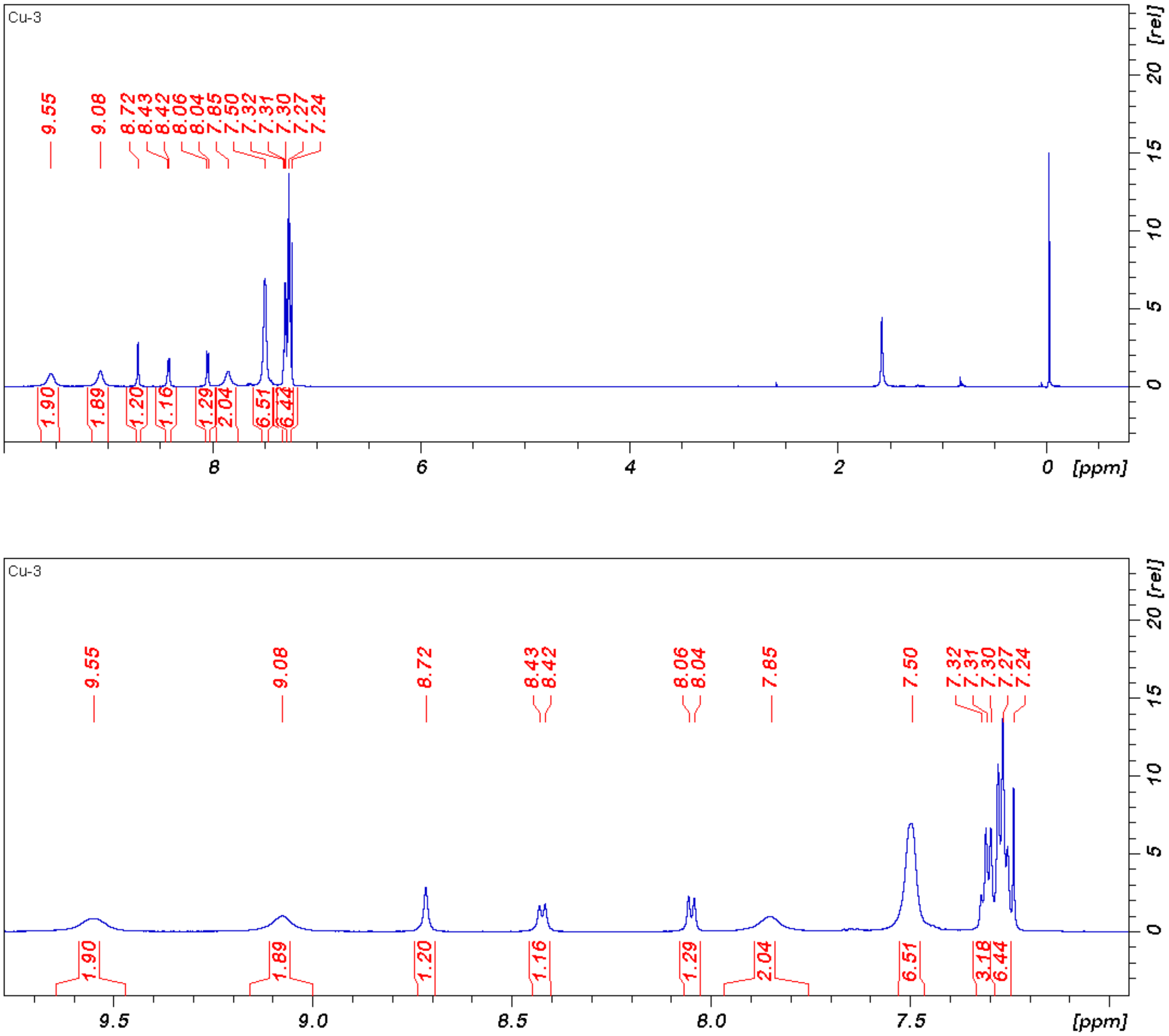

Figure S2. ^{1}H NMR of complex Cu-3.

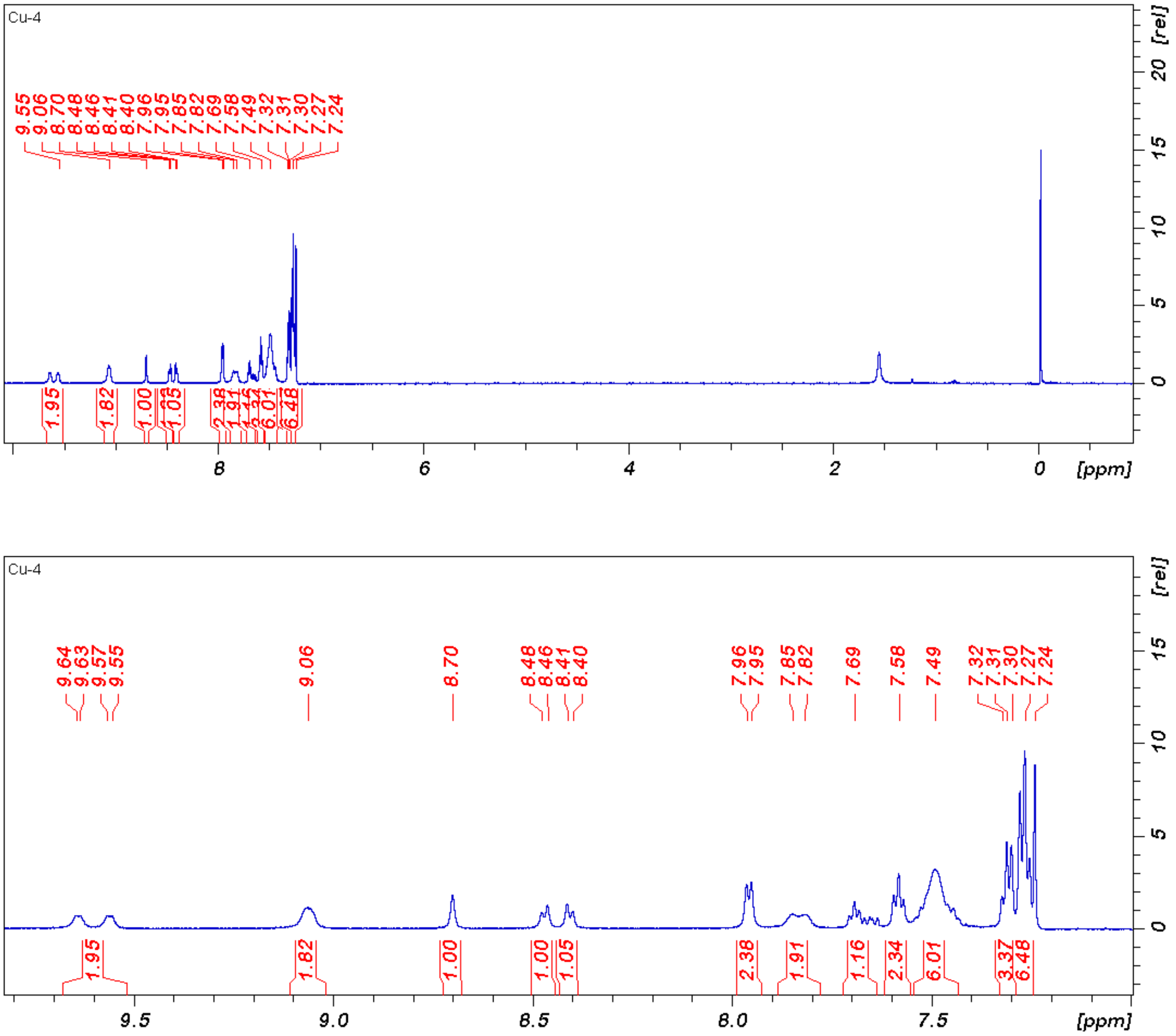

Figure S3. ^{1}H NMR of complex Cu-4.

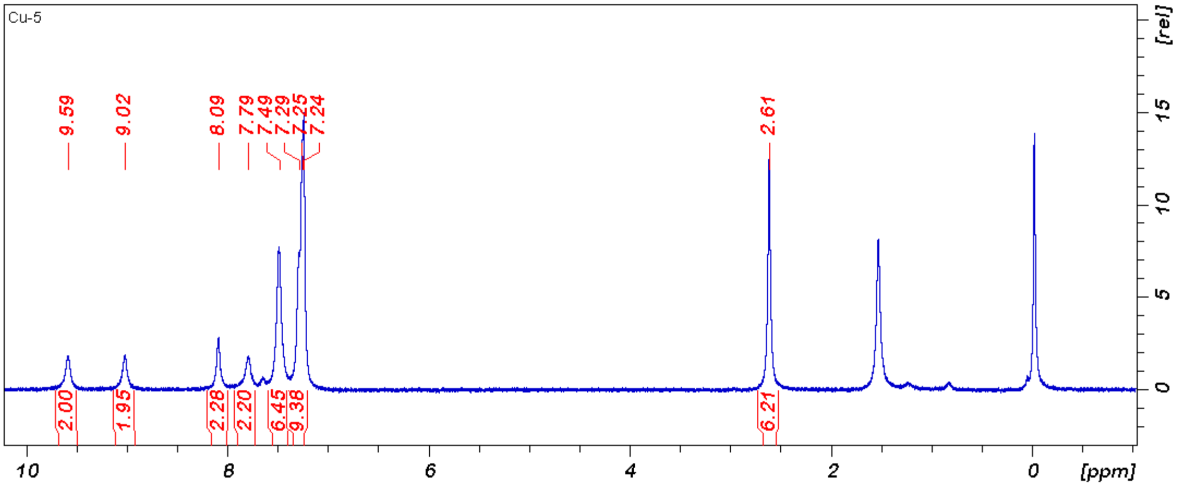

Figure S4. ^{1}H NMR of complex Cu-5.

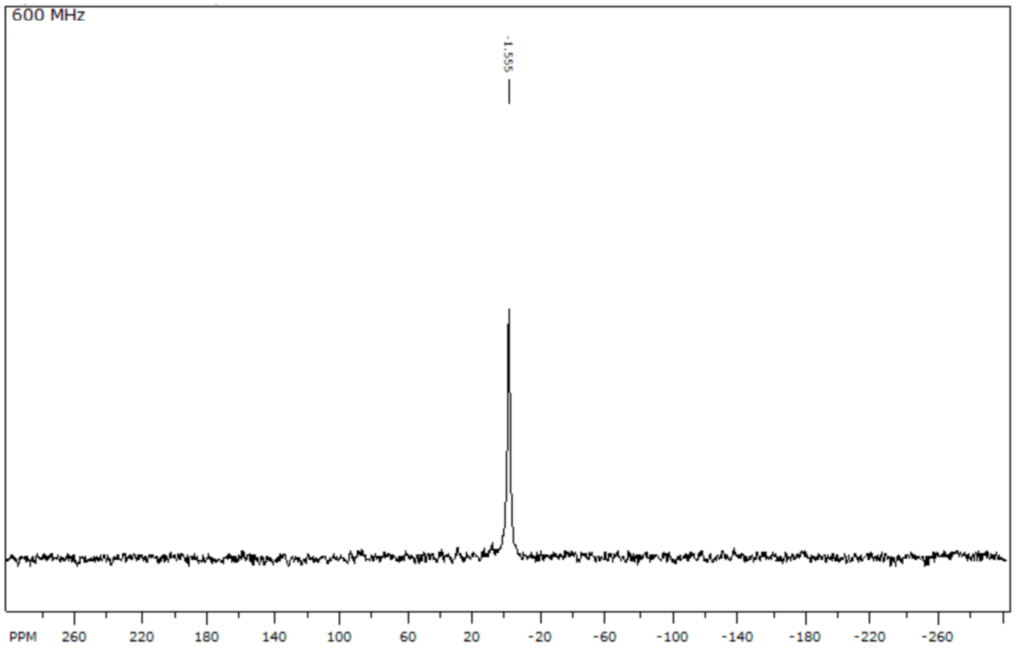

Figure S5. ^{31}P NMR of complex Cu-2.

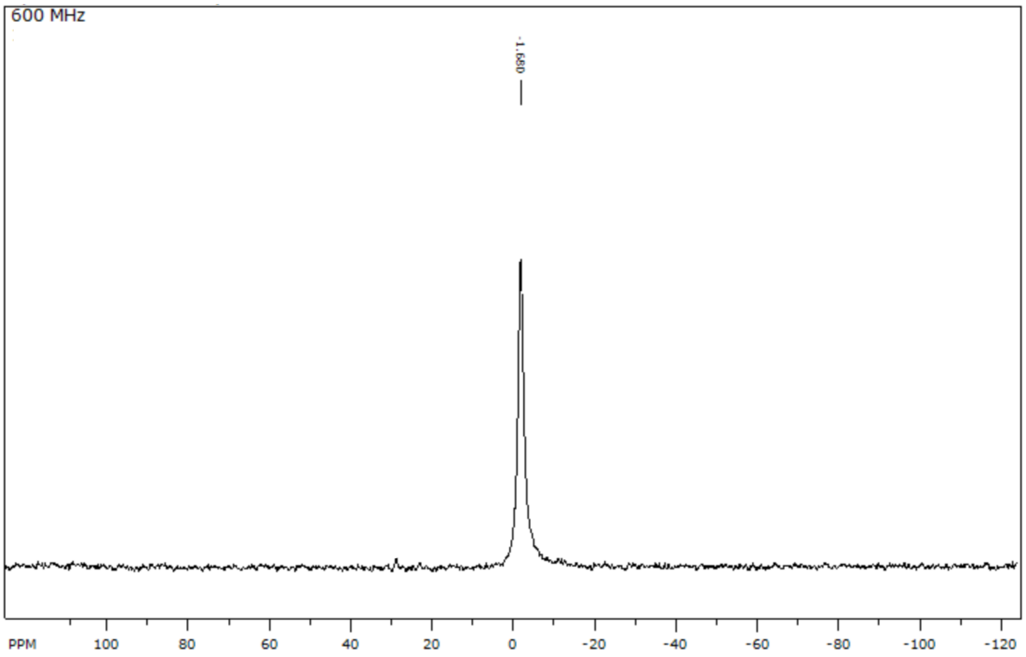

Figure S6. ^{31}P NMR of complex Cu-3.

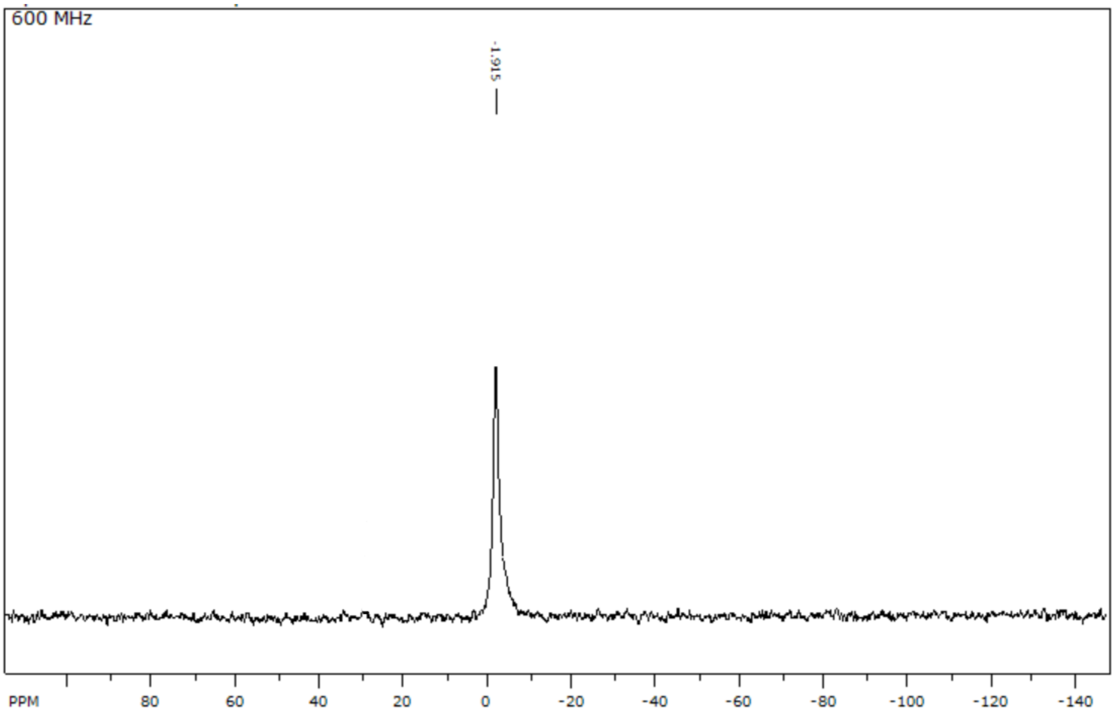

Figure S7. ^{31}P NMR of complex Cu-4.

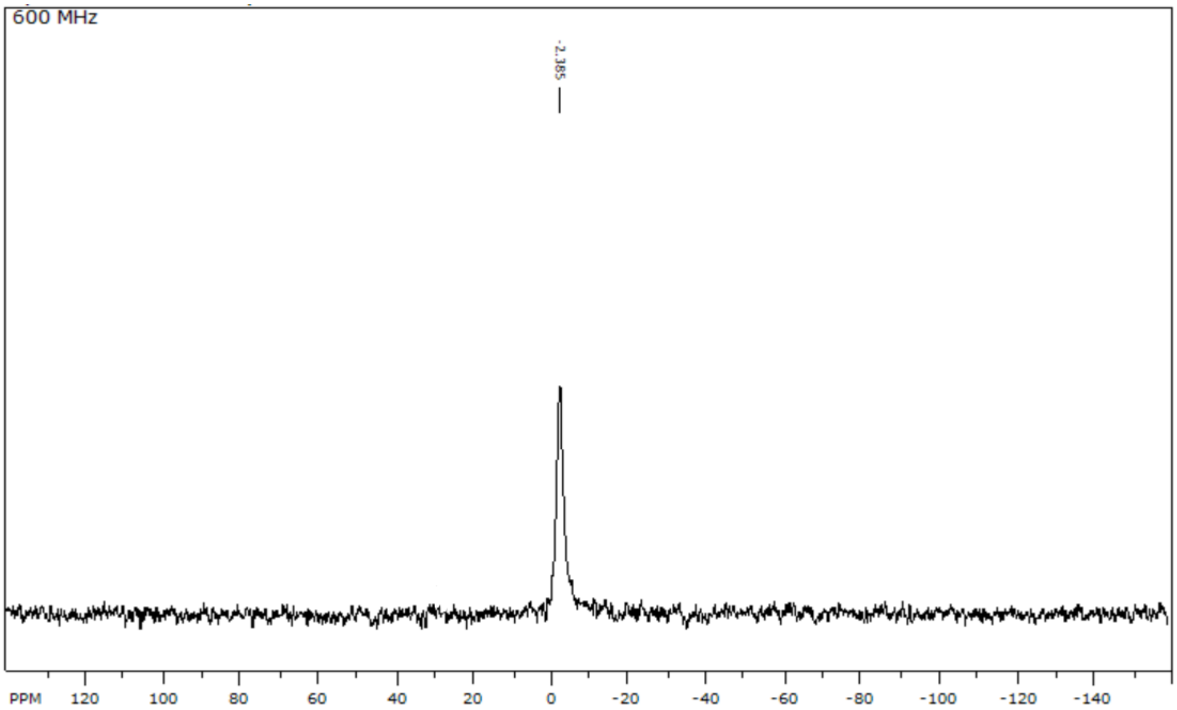

Figure S8. ^{31}P NMR of complex Cu-5.

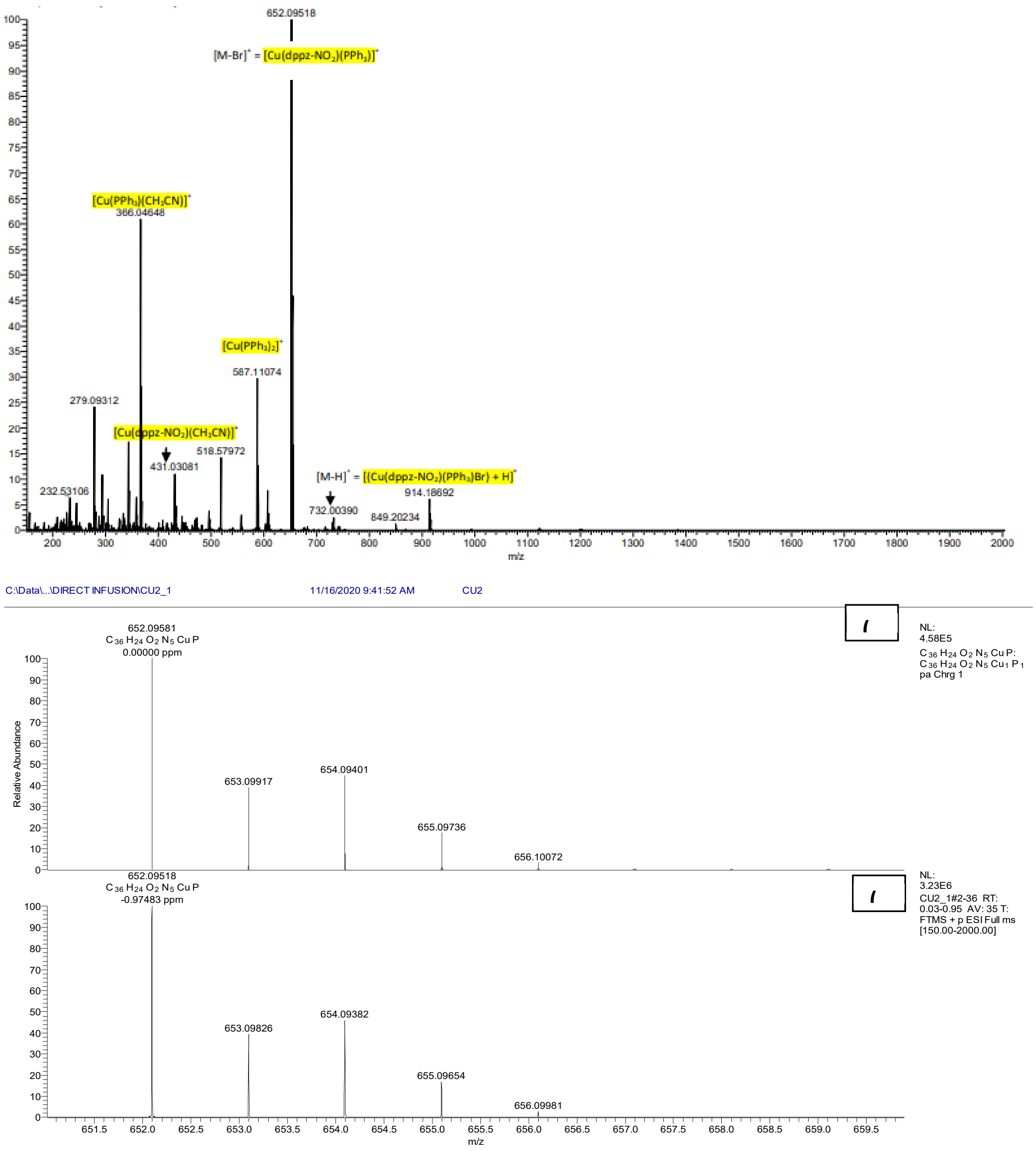

Figure S9. Low-resolution and high-resolution mass of complex Cu-2.

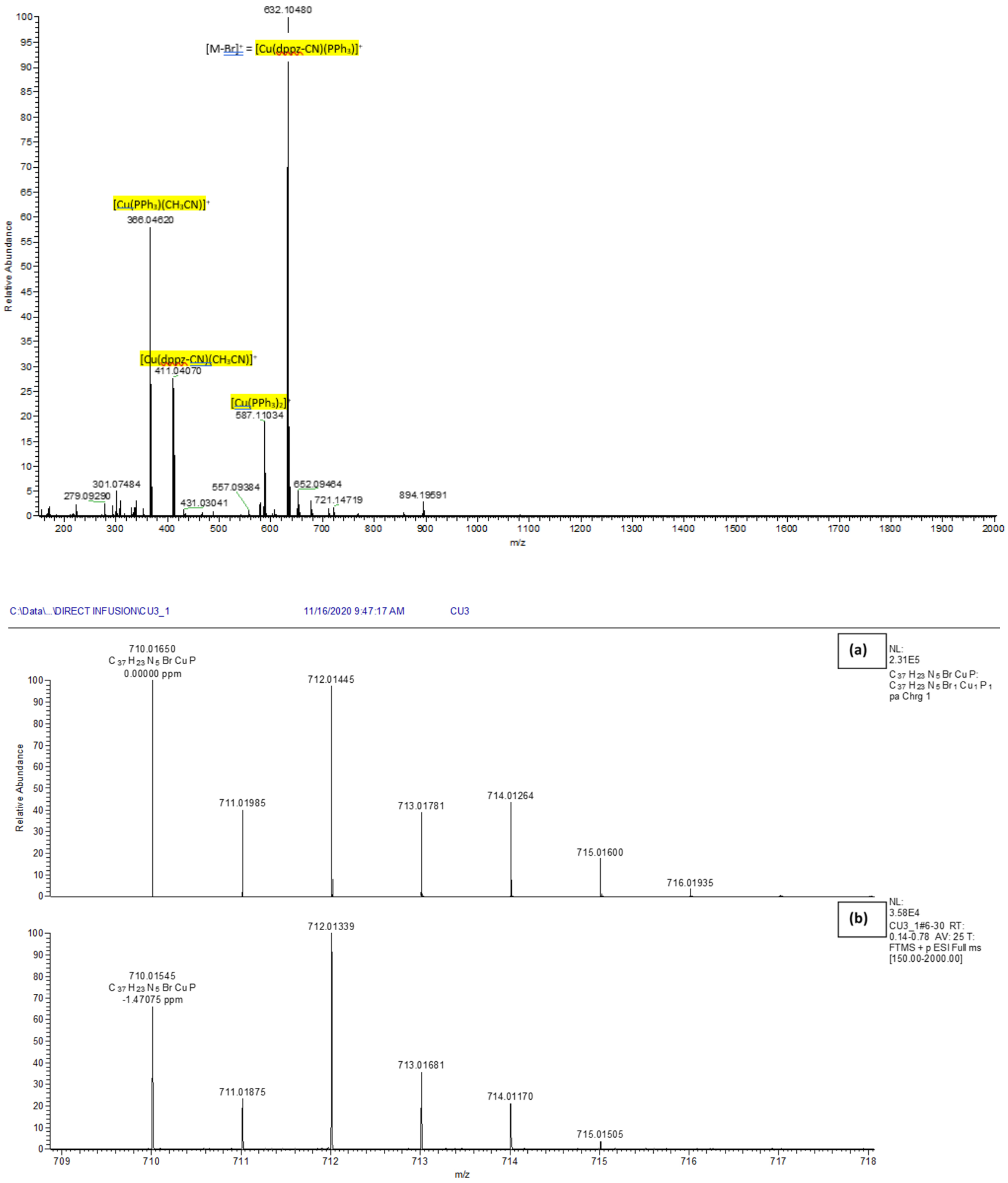

Figure S10. Low-resolution and high-resolution mass of complex Cu-3.

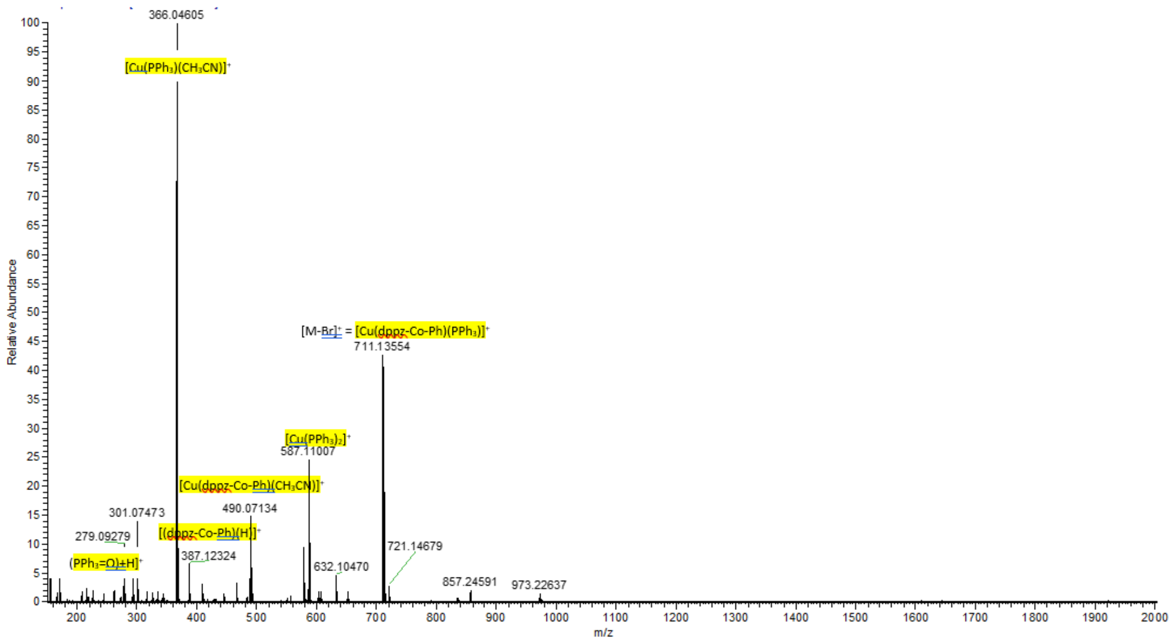

Figure S11. Low-resolution mass of complex Cu-4.

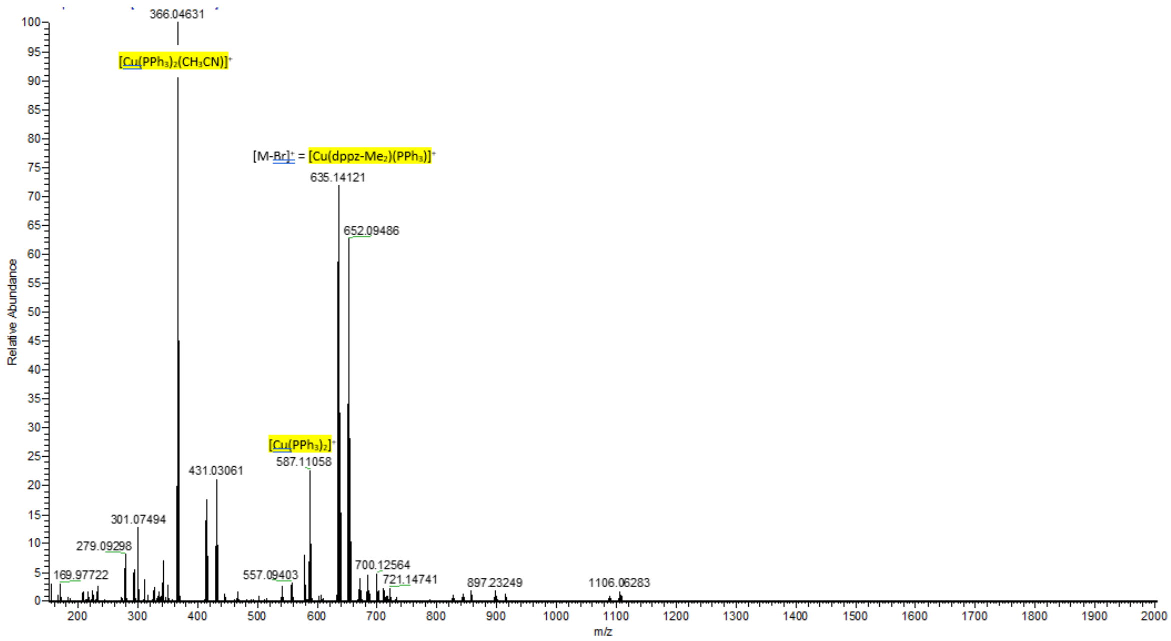

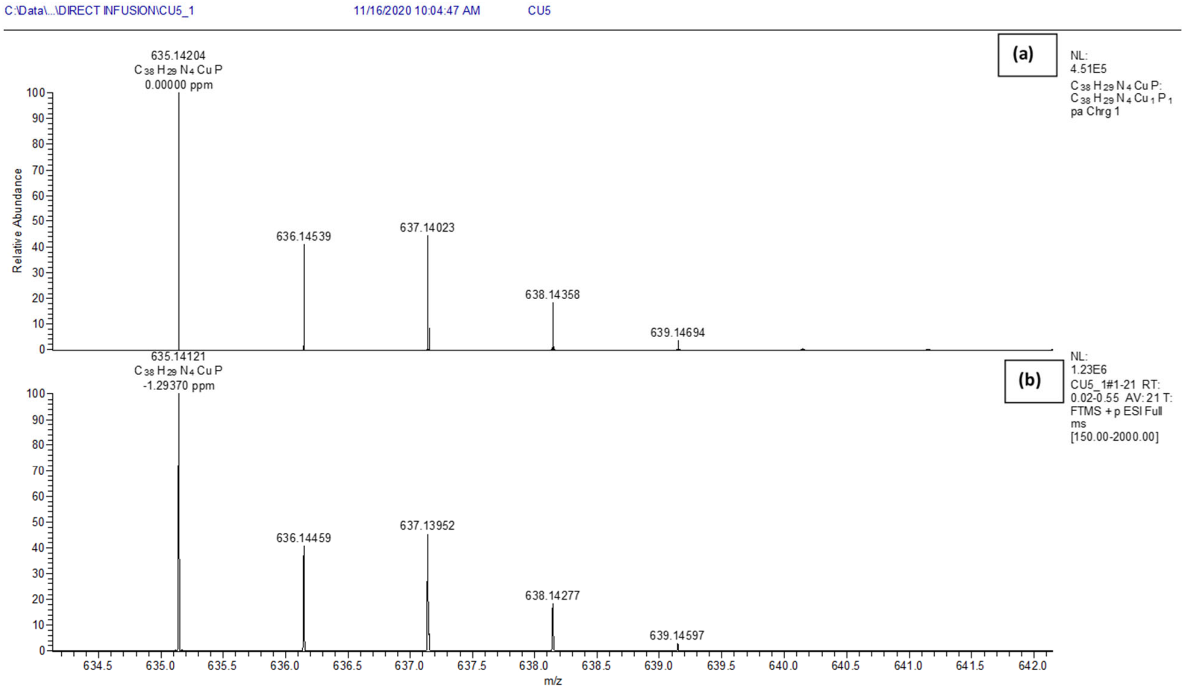

Figure S12. Low-resolution and high-resolution mass of complex Cu-5.

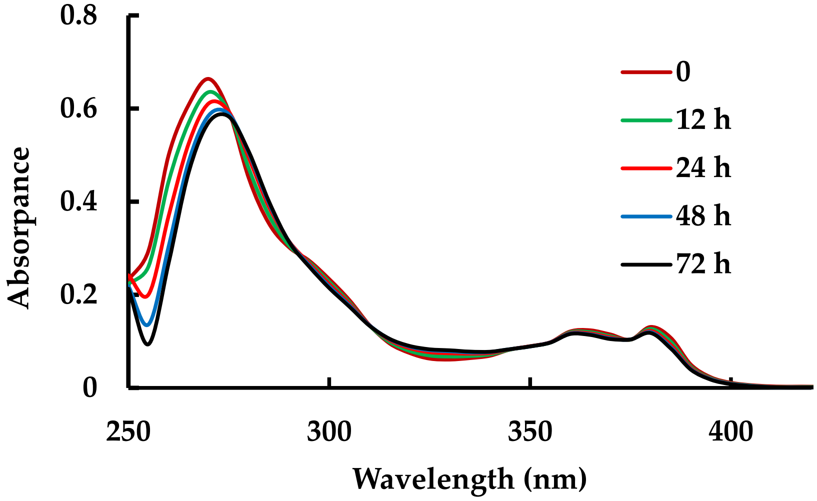

Figure S13. Overlaid UV-vis absorption of Cu-1 solution (1.3 × 10^{-5} M) in DMSO over several days, showing no sign of degradation.
